# Supplementary material for: Antiphospholipid Antibodies Occurrence in Acute SARS-CoV-2 Infection without Overt Thrombosis
Source: Biomedicines. 2023 Apr 22;11(5):1241. doi: 10.3390/biomedicines11051241 (PMC10215326; doi:10.3390/biomedicines11051241)
Supplement: Supplementary file 1 [file biomedicines-11-01241-s001.zip › biomedicines-2307925-supplementary.pdf]

Supplementary Materials

Supplementary Materials Table S1. Clinical manifestations during the severe acute respiratory syndrome coronavirus 2 (SARS-CoV-2) disease (COVID-19).

**Parameter**	**N=179**
Anosmia, n(%)	34/138 (19.0%)
Ageusia, n(%)	27/145 (15.1%)
Dyspnea, n(%)	67/108 (37.4%)
Cough, n(%)	114/59 (63.7%)
Odynophagia, n(%)	22/150 (12.3%)
Diarrhea, n(%)	36/136 (20.1%)
Vomiting, n(%)	24/148 (13.4%)
Loss of appetite, n(%)	37/135 (20.7%)
Headache, n(%)	55/117 (30.7%)
Myalgia, n(%)	75/97 (41.9%)
Fever, n(%)	106/66 (59.2%)
Shiver, n(%)	39/133 (21.8%)

Supplementary Materials Table S2. Comorbidities associated in the study group.

Parameter	N=179
Chronic renal disease, n(%)	15/160 (8.4%)
Diabetes, n(%)	48/127 (26.8%)
Arterial hypertension, n(%)	98/77 (54.7%)
Dyslipidemia, n(%)	44/131 (24.6%)
Obesity, n(%)	44/131 (24.6%)
Heart failure, n(%)	21/154 (11.7%)
Ischemic cardiac disease, n(%)	23/152 (12.8%)
Hepatic chronic disease, n(%)	15/160 (8.4%)
Chronic respiratory disease, n(%)	17/158 (9.5%)
Neoplasia history, n(%)	9/166 (5.0%)
At least two comorbidities, n(%)	88/87 (49.2%)

Supplementary Materials Table S3. Results of the biochemical tests in the patients included.

**Parameter**	**N=179**
Ferritin, ng/mL med(q1;q3) n=176	490.5 (239.0; 808.0)
Maximum ferritin, ng/mL med(q1;q3) n=176	536.5 (284.7; 917.0)
Leucocytes /µL, med(q1;q3)	6340 (4660; 8777)
Neutrophils /µL, med(q1;q3)	4320 (2920; 6840)
Lymphocytes /µL, med(q1;q3)	970 (710; 1460)
Minimum lymphocytes /µL, med(q1;q3)	780 (577; 1170)
CRP, mg/dL med(q1;q3)	38.7 (9.5; 85.3)
Maximum CRP, mg/dL med(q1;q3)	58.0 (20.0; 115.3)
Interleukin-6, pg/mL med(q1;q3) n=84	24.9 (11.2; 58.4)
Maximum Interleukin-6, pg/mL med(q1;q3) n=103	41.9 (14.6; 74.1)
Creatin-kinase, U/L med(q1;q3) n=175	92 (54; 175)
Troponin, pg/ml med(q1;q3) n=45	11.7 (7.2; 25.9)
Maximum troponin, pg/ml med(q1;q3) n=41	12.5 (7.36; 45.1)
NT pro-BNP, pg/mL med(q1;q3) n=95	336 (140; 984)
ALAT, U/L med(q1;q3) n=175	30.7 (19.4; 47.6)
Creatinine, mg/dL med(q1;q3)	0.84 (0.68; 1.08)
Albumin, g/dL med(q1;q3) n=141	3.9 (3.6; 4.2)
25OH-vitamineD, ng/mL med(q1;q3) n=143	23.0 (16.1; 33.4)
ALAT – alanine aminotransferase; CRP – C-reactive protein; NT pro-BNP – N-terminal pro b-type natriuretic peptide	

Supplementary Materials Table S4. Antiphospholipid antibodies and coagulation tests.

**Parameter**	**N=179**
Lupus anticoagulant, value mean±SD	1.52±0.24
Lupus anticoagulant<br>Negative <1.2, n(%)<br>Weakly positive 1.2 – 1.5, n(%)<br>Positive 1.5 – 2.0, n(%)<br>Strongly positive >2.0, n(%)	 11/172 (6.1%)<br>78/172 (43.6%)<br>75/172 (41.9%)<br>8/172 (4.5%)
Anticardiolipin antibodies IgM, MPL med (q1; q3)	8.71 (5.9; 13.7)
Anticardiolipin antibodies IgM, n(%)	17/174 (9.5%)
Anticardiolipin antibodies IgG, GPL med (q1; q3)	5.7 (3.7; 9.4)
Anticardiolipin antibodies IgG, n(%)	8/167 (4.5%)
Anti-beta2 glycoprotein 2 I beta IgG, GBU med (q1; q3)	2.7 (1.9; 4.1)
Anti-beta2 glycoprotein 2 I beta IgG, n(%)	3/172 (1.7%)
D-dimers, μg/mL med (q1; q3)	0.67 (0.37;1.12)
Maximum D-dimers, μg/mL med (q1; q3)	0.78 (0.48; 1.55)
Increased D-dimers, n(%)	105/178 (99.4%)
PT, sec mean±SD	14.0 (13.1; 15.0)
aPTT sec mean±SD	29.4 (26.9; 31.7)
Platelets x10^3 /μL, med (q1; q3)	221 (177; 303)
PDW,% mean±SD	11.9±3.1

aPTT – activated partial thromboplastin clotting time; PT – prothrombin time; PDW - platelet distribution width; SD – standard deviation.

Supplementary Materials Table S5. Correlations found between the antiphospholipid antibodies' levels.

	**LA**	**aCL IgM** (MPL)	**aCL IgG** (GPL)	**aB2GPI IgG** (GBU)
**LA**	-	p=0.107	p=0.852	p=0.141
**aCL IgM** (MPL)	p=0.107	-	p<0.001 rho=0.408	p<0.001 rho=0.282
**aCL IgG** (GPL)	p=0.852	p<0.001 rho=0.408	-	p<0.001 rho=0.436
**aB2GPI IgG** (GBU)	p=0.141	p<0.001 rho=0.282	p<0.001 rho=0.436	-

aCL – anticardiolipin antibodies; aB2GPI – anti-beta2 glycoprotein 2 I beta; LA – lupus anticoagulant. p-value; rho=Spearman's rank correlation coefficient

Supplementary Materials Table S6. Correlation of the antiphospholipid antibodies tested with the coagulation parameters.

	**D-dimers** (μg/mL)	**Maximum D-dimers** (μg/mL)	**PT** (sec)	**aPTT** (sec)	**Platelets** ($x10^3$ /μL)	**PDW** (%)
**LA**	p=0.016 rho=0.183	p=0.013 rho=0.190	p=0.203	p=0.001 rho=0.257	p<0.001 rho=-0.265	p=0.776
**aCL IgM** (MPL)	p=0.096	p=0.032 rho=0.172	p=0.031 rho=0.164	p=0.930	p=0.014 rho=0.187	p=0.785
**aCL IgG** (GPL)	p=0.067	p=0.061	p=0.011 rho=0.198	p=0.337	p=0.058	p=0.899
**aB2GPI IgG** (GBU)	p=0.038 rho=0.158	p=0.026 rho=0.170	p=0.001 rho=0.256	p=0.517	p=0.850	p=0.502

aCL – anticardiolipin antibodies; aB2GPI – anti-beta2 glycoprotein 2 I beta; aPTT – activated partial thromboplastin time; LA – lupus anticoagulant; aB2GPI – anti-beta2 glycoprotein 2 I beta; LA – lupus anticoagulant; PDW - Platelet Distribution Width; PT – prothrombin time.
p-value; rho=Spearman's rank correlation coefficient

Supplementary Materials Table S7. Bivariate analysis regarding the IgM anticardiolipin antibodies.

	**aCL IgM +**<br><br>17/174 (9.5%)	**aCL IgM –**<br><br>157/174 (87.7%)	**p-value**
Age, years mean±SD	65.4±8.3	59.3±14.8	0.112
Pulmonary involvement (CT), % med (q1;q3)	35.0 (10.0; 40.0)	30.0 (20.0; 40.0)	0.846
Anticardiolipin antibodies IgM, MLP med (q1; q3)	31.1 (6.0; 25.6)	7.71 (5.6; 11.6)	<0.001
Anticardiolipin antibodies IgG, GLP med (q1; q3)	12.4 (6.0; 25.6)	5.2 (3.6; 8.2)	<0.001
Anti-beta2 glycoprotein 2 I beta IgG, USG med (q1; q3)	3.8 (2.5; 4.4)	2.5 (1.9; 3.7)	0.052
D-dimers, µg/mL med (q1; q3)	0.9 (0.6; 1.6)	0.6 (0.3; 1.0)	0.072
PT, sec mean±SD	15.4±2.9	14.2±1.6	0.156
aPTT sec mean±SD	31.9±7.2	29.5±6.7	0.222
Ferritin, ng/mL med (q1;q3)	311.0 (206.5; 554.0)	518.5 (239.7; 871.0)	0.135
Lymphocytes /µL, med (q1;q3)	1020 (730; 1365)	960 (700; 1435)	0.853
Platelets x103 /µL, med (q1; q3)	231 (195; 368)	217 (176; 290)	0.174
PDW,% mean±SD	11.3±3.3	12.0±3.1	0.719
CRP,mg/dL med (q1;q3)	41.2 (11.6; 152.2)	41.5 (9.7; 85.5)	0.719
Interleukin-6, pg/mL med (q1;q3) n=84	45.7 (36.7; 226.9)	24.2 (9.8; 55.5)	0.029
ALAT, U/L med (q1;q3) n=175	34.5 (18.2; 48.6)	30.7 (20.2; 48.0)	0.931
Albumin, g/dL med (q1;q3) n=141	3.7 (3.3; 4.2)	3.9 (3.6; 4.3)	0.333
Creatin-kinase, U/L med (q1;q3) n=175	57.0 (41.0; 103.0)	105.0 (60.5; 188.5)	0.011
Troponin, pg/ml med (q1;q3) n=45	10.3 (6.9; 12.0)	13.5 (7.8; 40.3)	0.212
NT pro-BNP, pg/mL med (q1;q3) n=95	342.5 (160.0; 720.5)	336.0 (143.0; 800.0)	0.957
Creatinine, mg/dL med (q1;q3)	0.74 (0.59; 0.94)	0.86 (0.69; 1.10)	0.046
25OH-vitamine D, ng/mL med (q1;q3) n=143	18.0 (12.0; 30.6)	23.6 (16.1; 34.1)	0.140

aCL – anticardiolipin antibodies; ALAT - alanine aminotransferase; aPTT – activated partial thromboplastin time; aB2GPI – anti-beta2 glycoprotein 2 I beta; CT – computer tomography; LA – lupus anticoagulant; NT pro-BNP - N-terminal pro b-type natriuretic peptide; PDW - Platelet Distribution Width; PT – prothrombin time.

Supplementary Materials Table S8. Antiphospholipid antibodies expression in relation to the severity of the severe acute respiratory syndrome coronavirus 2 (SARS-CoV-2) disease (COVID-19).

**Parameter**	**Mild disease** N1=45	**Moderate disease** N2=62	**Severe disease** N3=72	***p*-value**
Lupus anticoagulant, rap med (q1; q3)	1.4 (1.2; 1.6)	1.5 (1.3; 1.7)	1.5 (1.4; 1.7)	0.027
Anticardiolipin antibodies IgM, MPL med (q1; q3)	10.3 (6.2; 15.5)	7.3 (6.5; 11.6)	9.1 (5.4; 12.9)	0.236
Anticardiolipin antibodies IgG, GPL med (q1; q3)	5.9 (4.0; 8.8)	5.5 (3.5; 8.3)	5.8 (4.0; 12.2)	0.358
Anti-beta2 glycoprotein 2 I beta IgG, GBU med (q1; q3)	3.1 (2.0; 5.6)	2.4 (1.8; 3.4)	2.6 (1.9; 4.6)	0.128

aCL – anticardiolipin antibodies; aB2GPI – anti-beta2 glycoprotein 2 I beta; GBU—microgram of IgG anti-beta2 glycoprotein I beta antibody per liter; GPL—microgram of IgG anticardiolipin antibody per liter; LA – lupus anticoagulant; MPL—microgram of IgM anticardiolipin antibody per liter
p-value by the Kruskal-Wallis test.

Supplementary Materials Table S9. Multivariate analysis for predictors of positive lupus anticoagulant.

Parameter	Exp (B)	95% CI		*p*-value
		lower	upper	
Anticardiolipin antibodies IgM, MPL med (q1; q3)	1.023	0.987	1.059	0.213
Anti-beta2 glycoprotein 2 I beta IgG, GBU med (q1; q3)	0.949	0.844	1.066	0.377
Ferritin, ng/mL med (q1;q3)	1.000	1.000	1.000	0.639
Lymphocytes /µL, med (q1;q3)	1.000	1.000	1.001	0.563
PDW,% mean±SD	0.961	0.852	1.084	0.518
**CRP, mg/dL med (q1;q3)**	**1.008**	**1.001**	**1.016**	**0.042**
D dimers, µg/mL med (q1;q3)	1.009	0.868	1.173	0.903
aPTT, sec mean±SD	1.036	0.973	1.102	0.269
Platelets x10^3 /µL, med (q1; q3)	1.000	1.000	1.000	0.047

aPTT – activated partial thromboplastin clotting time; PT – prothrombin time; PDW - platelet distribution width.
